# Investigation of Serum and Macular Carotenoids in Central Serous Chorioretinopathy

**DOI:** 10.3389/fmed.2022.805305

**Published:** 2022-04-01

**Authors:** Yuying Ji, Yuhong Gan, Yongyue Su, Yining Zhang, Miaoling Li, Lan Mi, Chengguo Zuo, Feng Wen

**Affiliations:** State Key Laboratory of Ophthalmology, Zhongshan Ophthalmic Center, Sun Yat-sen University, Guangdong Provincial Key Laboratory of Ophthalmology and Visual Science, Guangdong Provincial Clinical Research Center for Ocular Diseases, Guangzhou, China

**Keywords:** central serous chorioretinopathy, macular pigment optical density, macular pigment, serum lutein, serum zeaxanthin

## Abstract

**Purpose:**

This study aimed to evaluate serum lutein and zeaxanthin levels and macular pigment optical density (MPOD) in central serous chorioretinopathy (CSC).

**Methods:**

Fifty-four patients with acute CSC (28–56 years old; 44 men and 10 women) and 62 matched controls were enrolled. Serum lutein and zeaxanthin were measured using the high-performance liquid chromatography–tandem mass spectrometry (HPLC–MS/MS) method. MPOD was measured at 7° of eccentricity and reported in parameters as “max” and “mean” optical density (OD) (Visucam 200; Carl Zeiss Meditec). MPOD was re-measured in 9 patients whose subretinal fluid was absorbed.

**Results:**

The average max OD and the mean OD in CSC were 0.275 ± 0.047 d.u. and 0.098 ± 0.018 d.u., respectively, which were significantly lower than the control (*p* < 0.001). The average MPOD value in the unaffected eyes of patients with CSC was 0.298 ± 0.045 for max OD, 0.106 ± 0.017 for mean OD, and both were significantly lower compared with the affected eyes (*p* < 0.001 for max OD, *p* = 0.01 for mean OD). In the 9 follow-up patients, the decrease in MPOD was partially recovered. The mean serum level was 409.80 ± 182.52 ng/ml for lutein and 22.97 ± 12.23 ng/ml for zeaxanthin in patients with CSC. In controls, the mean serum level was 393.38 ± 202.44 ng/ml for lutein and 22.16 ± 10.12 ng/ml for zeaxanthin. The difference was not statistically significant (*p* = 0.649, *p* = 0.698, respectively).

**Conclusion:**

MPOD decreased within 7° of eccentricity in CSC without serum lutein and zeaxanthin changes. The decrease may be due to the subretinal fluid. Whether local oxidative stress is involved in CSC and the supplementation with lutein and zeaxanthin is helpful for CSC requires further investigation.

## Introduction

Of the over 1,000 carotenoids found in nature, only lutein and zeaxanthin and their metabolites (meso-zeaxanthin) are present in the human macula, and they are called macular pigments (1). Most of the macular pigments are located in the Henle fiber. Some of them are also distributed in the inner segment and rods out segment (2, 3). Owing to the function of filtering blue haze, lutein, and zeaxanthin are important antioxidant materials (4). Macular lutein and zeaxanthin must be acquired through dietary intake, as they are not synthesized endogenously (5). The combination and transportation of lutein and zeaxanthin from the ingested food matrix to the retina are characterized by a complex multistep process and the exact mechanism remains unknown, but retinal pigment epithelium (RPE) and choroid play a role in the process (6, 7).

Central serous chorioretinopathy (CSC) is a common disease characterized by serous retinal detachment (SRD) involving the macula (8). The typical findings are RPE leakage and SRD in fluorescein fundus angiography (FFA). It often affects young men and ranks fourth among non-surgical retinal diseases. The underlying mechanism of the disease is still under exploration, and increasing evidence has shown that choroidal and RPE dysfunction play an important role in pathogenesis (8, 9). Recently, some researchers have explored that oxidative stress may play a role in the pathogenesis of CSC (10–12). Sawa et al. (13) discovered that short-term lutein supplementation did not increase the MPOD values of patients with CSC, but could prevent the decline of MPOD in patients with CSC with lower plasma lutein.

The concentration of lutein/zeaxanthin/meso-zeaxanthin within the macula, or macular pigment optical density (MPOD), is used as a surrogate marker of macular health. Higher levels of MPOD are thought to preserve retinal tissue, specifically the layers containing photoreceptors in the fovea, and decreased macular pigment has been reported previously in macular disease, including age-related maculopathy, AMD, and CSC (13, 14). Moreover, depletion of carotenoids may result in lower MPOD and a greater risk of incident retinopathy and visual dysfunction. A previous study by Sasamoto et al. (15) reported decreased MPOD values in both chronic CSC eyes and fellow eyes using the two-wavelength autofluorescence spectrometry method without measuring systematic lutein and zeaxanthin concentrations. However, the relationship of serum lutein and zeaxanthin and MPOD evaluation using the one-wavelength reflectometry method has not been studied in Chinese patients with CSC with a relatively larger sample size.

The goal of the current study was to evaluate serum lutein and zeaxanthin levels and MPOD in acute Chinese patients with CSC. Investigation of the relationship of serum and macular carotenoids levels in patients with CSC may provide new perspectives and management of the disease.

## Materials and Methods

The study procedure was approved by the Ethics Committee of Zhongshan Ophthalmic Center of Sun Yat-sen University. The protocol was strictly conducted in accordance with the Declaration of Helsinki. After explaining the purpose and procedures of the study, all subjects signed a written informed consent.

### Subjects

Patients with treatment–naïve acute CSC were enrolled from outpatients in Zhongshan Ophthalmic Center. Healthy volunteers free of ocular manifestations enrolled from oral and poster advertisements around our hospital were included as the control group. They all underwent physical examination, including height, weight, and blood pressure. Body mass index (BMI) was calculated as a person’s weight in kilograms divided by the square of height in meters. Oral questionnaires were taken to investigate the course of the disease and smoking status (smokers were defined as smoking at least one cigarette a day for at least 6 months). Ophthalmic examinations were performed in all patients with CSC, including visual acuity, slit-lamp biomicroscopy, direct ophthalmoscopy, color fundus photography, and fundus fluorescence angiography (FF 450 plus, Carl Zeiss, Germany). Visual acuity was required using the Early Treatment Diabetic Retinopathy (ETDRS) chart and recorded in the logarithm of the minimum angle of resolution (logMAR) format.

The inclusion criterion for acute CSC was focal SRD involving macula with one or more leakage in FFA within 3 months.

Patients with corneal disease, cataracts, glaucoma, and other fundus diseases such as rhegmatogenous retinal detachment, choroidal neovascularization, polypoidal choroidal vasculopathy (PCV), drusen, retinal vein occlusion, diabetic retinopathy, Vogt–Koyanagi–Harada, optic disc pit, and scleritis were excluded. Patients with trauma history or surgery within 1 month were also excluded.

For both patients and control participants, the general exclusion criteria were as follows: uncontrolled hypertension (systolic pressure was consistently greater than 140 mm Hg or when diastolic pressure was consistently 90 mm Hg or more), hyperlipidemia (total cholesterol more than 5.18 mmol/L, triglyceride more than 1.7 mmol/L), medical history that might influence the absorption of carotenoids such as lutein supplementation, and special diet habits such as vegetarian.

We also prospectively evaluated the change in MPOD during the course of the disease. Among the patients with acute CSC, patients without medication or laser treatment were followed up in a 3-month period using optical coherence tomography (OCT) (Spectralis, Heidelberg Engineering, Germany). Only 9 patients achieved spontaneous absorption of subretinal fluid. MPOD values were measured at baseline and the latest visits of these 9 patients.

### Macular Pigment Optical Density Measurement

Macular pigment optical density was detected using the one-wavelength reflectometry method (Visucam 200; Carl Zeiss Meditec) as previously described (16). All subjects’ pupils were dilated to a minimum diameter of 7 mm using 1% tropicamide before measurement. First, a fundus color photography in 45° was taken. Then, the MPOD photograph was measured in the 30°-field with flash intensity set to 12. Software in the instrument calculated the MPOD value in a 7°-eccentricity. The parameters included max optical density (OD) and mean OD. Max OD refers to the maximum OD of macular pigment carotenoids and the mean OD refers to the mean OD of the macular pigment carotenoids in relation to the surface area.

### Serum Analysis

We used high-performance liquid chromatography–tandem mass spectrometry (HPLC-MS/MS) method (Shimadzu LC20AD—API 3200MD TRAP) to assay the serum levels of lutein and zeaxanthin. Antecubital vein peripheral blood samples were obtained in the morning after an overnight fast. Serum was separated after centrifugation at 2,000 *g* for 10 min at 4°C and stored at –80°C for later analysis. A 100 μl sample was mixed with 300 μl of chloroform–methanol solution and 300 μl of pure water for 1 min on a shaker. Then they were refrigerated and centrifugated at 13,200 *g* for 8 min. After that, the upper organic phase layer was immediately evaporated to dryness under a stream of nitrogen by using a sample concentrator in an eppendorf (EP) tube. The water phase was mixed with 300 μl N-hexane on a shaker for 1 min and was refrigerated by centrifugation at 13,200 *g* for 8 min. The upper organic phase was placed in the previous EP tube and evaporated to dryness. These dried samples were reconstituted in 100 μl methanol, and 50 μl was used for HPLC analysis.

### Statistical Analysis

SPSS software, version 19.0 (SPSS Inc., Chicago, IL, United States) was used for the analysis. The chi-square test was used to compare the sex and the smoking status between patients with CSC and controls. The independent *t*-test was used to compare the difference of age, visual acuity, and MPOD parameters between patients with CSC and controls. Paired *t*-test was used to compare visual acuity and MPOD differences between affected and unaffected eyes of patients with CSC, and in patients who had been followed up with the previous measurements. Pearson correlation was used to assess the association between MPOD value and visual acuity. A *p*-value less than 0.05 was considered to be statistically significant.

## Results

A total of 54 patients with acute CSC and 62 volunteers who met the criteria were enrolled in the study. Table 1 shows the general characteristics of patients with CSC and controls. Age, sex, BMI, and smoking status were not significantly different between the groups. In patients with CSC, the mean serum level of lutein was 409.80 ± 182.52 ng/ml and 22.97 ± 12.23 ng/ml for zeaxanthin. In normal controls, the mean serum level was 393.38 ± 202.44 ng/ml for lutein and 22.16 ± 10.12 ng/ml for zeaxanthin. The serum levels of lutein and zeaxanthin were not significantly different between the CSC group and the control group (*p* = 0.649, *p* = 0.698, respectively).

**TABLE 1:** General characteristics and serum L/Z levels in all the participants.

Characteristics	Acute CSC cases	Control	*P*-value
Number	54	62	
Sex (Male/Female)	44/10	50/12	0.920
Age (Mean ± SD) (Range)	45.11 ± 6.28 (28–56)	46.61 ± 8.94 (27–62)	0.304
BMI (Mean ± SD)	22.93 ± 2.44	22.83 ± 2.43	0.820
Smoking status (Yes/No)	32/22	40/22	0.560
Serum L (ng/ml)	409.80 ± 182.52	393.38 ± 202.44	0.649
Serum Z (ng/ml)	22.97 ± 12.23	22.16 ± 10.12	0.698

*BMI, body mass index; L, lutein; Z, zeaxanthin.*

The average MPOD value in patients with acute CSC was 0.275 ± 0.047 d.u. for max OD and 0.098 ± 0.017 d.u. for mean OD. In the control group, the average MPOD value was 0.348 ± 0.040 d.u. for max OD and 0.129 ± 0.022 d.u. for mean OD. Independent *t*-test showed that patients with acute CSC had lower MPOD values than controls, and the differences were significant (*p* < 0.001).

The average MPOD value in the unaffected eyes of patients with CSC was 0.298 ± 0.045 for max OD, and 0.106 ± 0.017 for mean OD, and both were significantly reduced compared with the affected eyes (*p* = 0.018 for max OD, *p* = 0.015 for mean OD, paired *t*-test).

In terms of visual acuity, the mean logMAR visual acuity in the affected eyes of patients with CSC was 0.137 ± 0.216, in unaffected eye of patients with CSC was 0.044 ± 0.134, and in the control group was –0.016 ± 0.064. The difference was significant. (Affected eyes vs. unaffected eyes in CSC patients, *p* < 0.001, paired *t*-test. Affected eye in patients with CSC vs. control group, *p* < 0.001, independent *t*-test).

**FIGURE 1:**
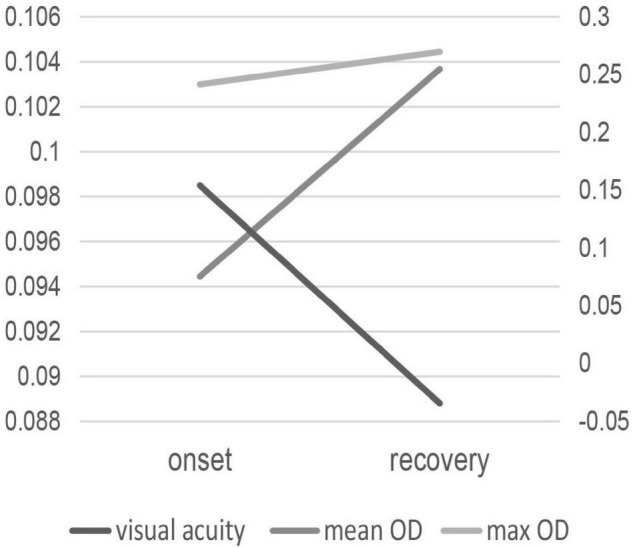
The change of MPOD values and visual acuity in the 9 patients with subretinal fluid absorbed at disease onset and recovery.

In the 9 patients whose subretinal fluid resolved spontaneously at the 3-month follow-up time, MPOD values showed a rising tendency and was significant correlated with visual acuity at disease onset, as shown in Figure 1. Table 2 shows the correlation between MPOD value and visual acuity in the 9 follow-up patients during the period of onset and recovery of the disease. Max OD was 0.241 ± 0.045 d.u. at disease onset and 0.270 ± 0.048 d.u. at the recovery time with a marginally significant difference (*p* = 0.043, paired *t*-test). For mean OD, the value was 0.094 ± 0.018 d.u. at disease onset and 0.104 ± 0.018 d.u. at recovery time, the difference was insignificant (*p* = 0.075, paired *t*-test). At the disease onset, visual acuity was significantly correlated with MPOD, for max OD, rs = –0.771, *p* = 0.015, for mean OD, rs = –0.813, *p* = 0.008. The average BMI value was 22.61 ± 2.64 in these 9 patients with 3 patients smoking, with no differences compared with the other patients.

**TABLE 2:** Correlation between MPOD values and visual acuity in the 9 patients with CSC at disease onset and recovery.

Time		MPOD value	Visual acuity (logMAR)	*R*	*P*
Onset	Max OD	0.241 ± 0.045	0.122 ± 0.180	−0.771	0.015
	Mean OD	0.094 ± 0.018		−0.813	0.008
Recovery	Max OD	0.270 ± 0.048	−0.035 ± 0.122	−0.630	0.069
	Mean OD	0.104 ± 0.018		−0.451	0.224

**FIGURE 2:**
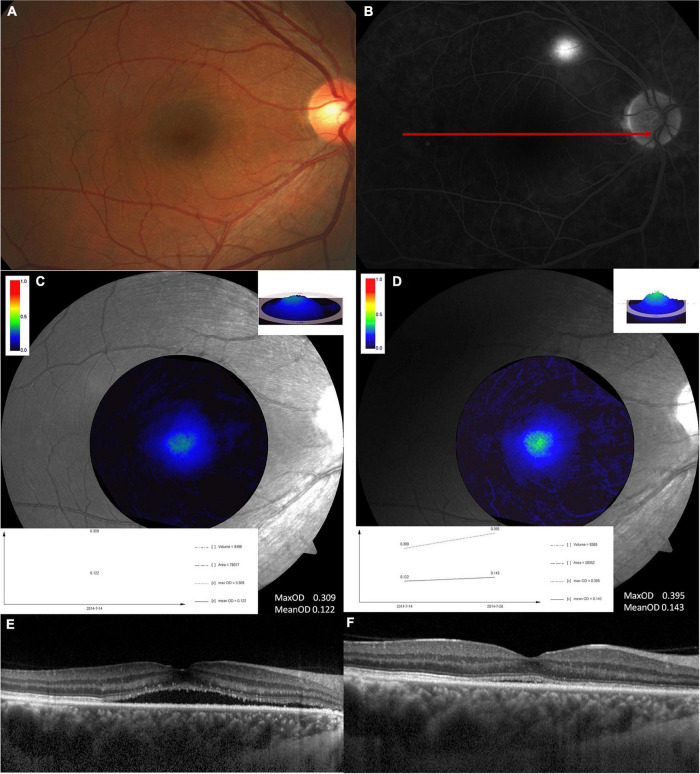
A typical example showing MPOD changes at the disease onset and the recovery time. This is a 45-years-old male. **(A)** Fundus image and **(B)** fluorescence fundus angiography image at the disease onset. The second row is the MPOD value analyzed at disease onset **(C)** and recovery time **(D)**. Both max OD and mean OD increased at the recovery time compared with that at disease onset. The exact MPOD value is marked in the picture. The third row shows the optical coherence tomography findings at disease onset **(E)** and the recovery time **(F)**.

Figure 2 shows a typical case of MPOD changes at disease onset and the recovery time and it indicates the MPOD increased when the disease was recovered.

## Discussion

Our study demonstrated that MPOD values were decreased in patients with acute CSC using the one-wavelength reflectometry method. The decreased MPOD value in CSC patients was similar to a previous study carried out in Japan including 70 patients with chronic CSC and 41 patients with acute CSC using the autofluorescence method (15). What caused the reduction of MPOD in patients with CSC remains unknown. The possible reasons are discussed as follows. First, recent studies have shown that the antioxidative process might be involved in the pathogenesis of CSC (10–12). The blood flow regulation of choroidal vessels may be affected by nitric oxide and free radicals. In this way, carotenoids could be overconsumed locally because of their antioxidation function. As a result, MPOD is decreased.

Second, abnormalities of choroidal vessels and RPE in patients with CSC may account for this result. The majority of macular pigments are distributed in the outer plexiform layer without capillaries. Carotenoids are transported to the retina by blood circulation (7, 17). Through choroidal circulation and RPE, carotenoids are captured and accumulated in the neurosensory retina (18, 19). Choroidopathy and epitheliopathy all take part in the pathogenesis of CSC. The patients with CSC have pachychoroid compared with normal people in both affected and unaffected eyes due to the dilated large choroidal vessels (20–22). Various studies have shown that dysregulation of choroidal blood flow exists in patients with CSC (23, 24). These abnormalities would influence the transportation and capture of carotenoids.

Our study also found that MPOD values in the unaffected fellow eyes of patients with CSC were significantly greater than the affected eyes. At the disease onset time, a significant correlation was found between visual acuity and MPOD. Furthermore, in the follow-up of 9 patients, MPOD values partially recovered with the same tendency of visual acuity. The same change tendency of MPOD and visual acuity suggest that MPOD was an indicator of macular health. Besides the function of blue light filtering, lutein is reported to protect photoreceptor cells (25, 26) and is closely related with visual functions such as photostress recovery, glare disability, and contrast sensitivity (27). The results also implied that subretinal fluid may have an impact on the measurement of MPOD in patients with acute CSC. The fluid underlying the neurosensory retina could cause damage to RPE and photoreceptors, thus influencing the capture and accumulation of carotenoids (2, 7). In addition, subretinal fluid may affect lutein transport from the choroid to the retina and cause a shortage of macular pigment in the retina. The methodologic artifacts of one-wavelength reflectometry should also be considered as a bias of these results. No other obvious difference was found in BMI and smoking status between these 9 and the other patients. This may be partially due to the limited number.

Patients with CSC had no differences in serum lutein concentration compared with healthy subjects in the current study. To our knowledge, serum lutein and zeaxanthin levels have not been measured in a relatively larger sample size of patients with CSC. Sawa et al. (13) measured plasma lutein concentrations in patients with CSC and healthy subjects. The results showed that patients with CSC had a lower plasma lutein concentration than healthy subjects, which is inconsistent with our findings. The following reasons may provide a plausible explanation. We included more patients with CSC and healthy participants and only focused on patients with acute CSC while in Sawa’s study, patients with chronic CSC were involved.

Although CSC is a common disease in ocular fundus outpatient department, many mysterious about its pathophysiology have not been uncovered for decades. Previous studies had shown that oxidative stress may play a role in CSC (12). Antioxidant supplementation including lutein may improve best corrected visual acuity in patients with chronic CSC (28). The local carotenoid decreases without serum change in patients with acute CSC provides us new aspects in understanding this disease. More attention should be paid to the local changes when it comes to antioxidant supplementation in CSC.

There are some limitations of our study. Choroidal thickness, central retinal thickness, oxidative parameters, and visual function tests such as contrast sensitivity were not examined, which limit our understanding of the relationship between lutein and zeaxanthin levels and the pathogenesis of CSC. The follow-up duration was limited to 3 months and the patients with follow-up were limited to 9 patients without serum carotenoid re-measurement. Information of patients who do not have fluid resorption was lacking, which could help us to investigate the clinical use of MPOD in CSC further. Randomized controlled interventional studies with more patients and longer follow-up time would be helpful to understand the relationship between carotenoid and CSC.

Our research measured serum lutein and zeaxanthin levels and MPOD values in patients with CSC and found that MPOD was reduced in patients with CSC using the one-wavelength reflectometry method. Serum lutein and zeaxanthin levels did not vary significantly in patients with CSC and healthy controls. The local difference in macular pigment may be affected by subretinal fluid. Whether local oxidative stress is involved in CSC and whether supplementation with carotenoids is helpful for the recovery of CSC requires further investigation.

## Data Availability Statement

The raw data supporting the conclusions of this article will be made available by the authors, without undue reservation.

## Ethics Statement

The studies involving human participants were reviewed and approved by the Ethics Committee of Zhongshan Ophthalmic Center of Sun Yat-sen University. The patients/participants provided their written informed consent to participate in this study. Written informed consent was obtained from the individual(s) for the publication of any potentially identifiable images or data included in this article.

## Author Contributions

YJ, YG, YS, and YZ contributed to the study design and data collection, analysis, and interpretation. LM, ML, CZ, and FW contributed to the data collection and data interpretation. All authors revised the manuscript and gave final approval for submission.

## Conflict of Interest

The authors declare that the research was conducted in the absence of any commercial or financial relationships that could be construed as a potential conflict of interest.

## Publisher’s Note

All claims expressed in this article are solely those of the authors and do not necessarily represent those of their affiliated organizations, or those of the publisher, the editors and the reviewers. Any product that may be evaluated in this article, or claim that may be made by its manufacturer, is not guaranteed or endorsed by the publisher.
